# Assessing the effectiveness of seasonal malaria chemoprevention on malaria burden among children under 5 in northern Benin: a statistical modelling approach

**DOI:** 10.1136/bmjph-2025-004335

**Published:** 2026-07-27

**Authors:** Roland Christel Sonounameto, Barikissou Georgia Damien, Timóteo A Sambo, William Houndjo, Julien Aissan, Rock Aikpon, Codjo Dandonougbo, Romain Glèlè Kakaï, Emilie Pothin

**Affiliations:** 1 University of Basel, Basel, Switzerland; 2 Swiss Tropical and Public Health Institute, Allschwil, Switzerland; 3 Research and Innovation Centre, African Institute for Mathematical Sciences, Kigali, Rwanda; 4 University of Abomey-Calavi, Laboratoire de Biomathématiques et d’Estimations Forestières, Abomey-Calavi, Benin; 5 University of Abomey-Calavi, Centre de Formation et de Recherche en matière de Population, Cotonou, Benin; 6 Ministry of Health, National Malaria Control Programme, Cotonou, Benin

**Keywords:** Public Health, Incidence, statistics and numerical data

## Abstract

**Introduction:**

Children under 5 in northern Benin remain highly vulnerable to malaria infection. Seasonal malaria chemoprevention (SMC), implemented since 2019, shows promise in reducing this burden. However, current malaria funding cuts threaten its future implementation putting additional children’s lives at risk. Generating evidence about SMC’s effectiveness is therefore crucial to support continued implementation.

**Methods:**

A quasi-experimental analysis evaluated SMC’s impact on malaria morbidity in children under 5 during 2019–2022. Routine malaria indicators retrieved from the Health Management Information System from six SMC-treated health zones (HZs) were compared against synthetic counterfactuals constructed using nine eligibles untreated HZs. Synthetic control methods identified control HZs best matching intervention HZs on pre-intervention (2017–2018) characteristics (rainfall, temperature, relative humidity), with pre-intervention fit assessed using root mean square prediction error. Generalised linear mixed models then estimated SMC effects on uncomplicated and severe malaria incidences, accounting for reporting bias, other interventions and key environmental factors.

**Results:**

Following SMC implementation, severe malaria incidence decreased by 41% in the intervention group (incidence rate ratio (IRR): 0.59, 95% CI 0.44 to 0.79) but increased by 26% in the control group (IRR: 1.30, 95% CI 0.97 to 1.63). Although uncomplicated malaria incidence rose in both groups, the increase was significantly lower in the SMC group (2.49%) compared with the control (16.3%). The ratio of IRR in the intervention versus control HZs was 78% (95% CI 63% to 96%, p=0.004) for uncomplicated malaria and 47% (95% CI 30% to 74%, p<0.001) for severe malaria, confirming a protective effect of SMC on both outcomes.

**Conclusions:**

These findings strongly support continuing SMC implementation in northern Benin and its geographical expansion to eligible HZs not yet covered.

**WHAT IS ALREADY KNOWN ON THIS TOPIC**

- Seasonal malaria chemoprevention (SMC) is a safe and effective intervention for preventing clinical malaria cases in children aged 6–59 months and has been implemented in northern Benin since 2019. However, current malaria funding cuts threaten its future implementation, putting additional children’s lives at risk.
- Routine Health Management Information System (HMIS) data provides opportunities for impact evaluation.

**WHAT THIS STUDY ADDS**

- This is one of the first studies to evaluate SMC impact in Benin using routine HMIS data with a robust quasi-experimental design comparing intervention and control health zones (HZs).
- The intervention was associated with a 53% reduction in severe malaria incidence (incidence rate ratio (IRR) intervention HZs vs control HZs=47, 95% CI 30 to 74) and a 22% reduction in uncomplicated malaria incidence (IRR intervention HZs vs control HZs=78, 95% CI 65 to 98), indicating significant protective effects against both outcomes.

**HOW THIS STUDY MIGHT AFFECT RESEARCH, PRACTICE OR POLICY**

- Routine HMIS data can be effectively used to measure malaria interventions impact like SMC on malaria morbidity when combined with appropriate statistical methods and control comparisons.
- The evidence of significant protective effects against both severe and uncomplicated malaria strongly supports continued SMC implementation in northern Benin and can be used to advocate for sustained funding.

## Introduction

Malaria remains a severe public health concern, causing the most harm in sub-Saharan Africa, especially among children under 5 years old. In 2023, Africa accounted for 95% of the global malaria deaths, with 76% occurring in children under 5 years of age,^1^ mainly during high rainfall periods.^2^ Because of the seasonality in malaria transmission in most African countries,^3–5^ the WHO has recommended seasonal malaria chemoprevention (SMC) to prevent infection and reduce the burden in young children.^6^ The WHO guidelines state that SMC is given to all children under the age of 5 for three to four continuous months during the rainy season. This usually involves 1 monthly intake of sulfadoxine-pyrimethamine (SP) and three daily doses of amodiaquine (AQ).^7^ SMC is a safe and very effective way to prevent malaria in children under 5,^8^ and even in children up to 10 years old.^9 10^ A systematic evaluation of studies on SMC efficacy in sub-Saharan Africa indicated protective efficacy against clinical malaria ranging from roughly 60−85% over 28 days.^7^ Although strong evidence supports the efficacy of SMC, assessing its effectiveness in real-world conditions remains essential for developing context-specific implementation strategies, particularly given limited resources. Real-world protective effectiveness falls below trial predictions,^11–14^ highlighting the need for evaluations beyond randomised controlled trials.^15^ Additionally, improved health management information systems now facilitate country-wide impact assessment, enabling timelier, data-driven decisions and more precise targeting of interventions based on real-time evidence. With stronger data, the evaluations not only assess intervention effectiveness but can also identify implementation barriers^11 15^ and optimise resource allocation.^15^

In northern Benin, malaria disproportionately affects children under 5^16^ due to highly seasonal transmission patterns.^17 18^ Periods of high rainfall and lower temperatures constitute peak transmission seasons.^18^ Benin’s National Malaria Control Programme (NMCP) introduced SMC in 2019 as a response to the country’s malaria burden. The programme began in two health zones (HZs), then gradually expanded—reaching four HZs in 2020 and six HZs by 2021. This implementation followed the eligibility criteria established by the WHO.^19^ A 2023 post-campaign cross-sectional analysis revealed average effective coverage of 87.58% and full 12-dose coverage of 59.8% across all six HZs.^20^ Research indicates that high effectiveness correlates with extensive coverage, type of delivery strategies and strong adherence.^21–25^ Despite efforts to improve SMC coverage in Benin,^19^ coverage decreases with subsequent dosages. It varies from one HZ to another,^20^ requiring effectiveness assessments to understand the real impact of such a programme and informing future decision-making.^20 26^ HMIS databases are a rich and readily available source of data for the evaluation of public health interventions^27 28^ such as SMC. Routine data from Benin’s health system is collected through the HMIS, which relies on the pyramidal organisation of the country’s health system. These data are collected using various tools, including registers, forms, cards and booklets. Among these tools, curative care registers constitute the fundamental element of epidemiological surveillance. They provide information on patients’ details (name and surname, sex, age, etc), symptoms, biological examination results, diagnosis and treatment. This information is compiled monthly into an epidemiological report. Regarding malaria, these epidemiological reports are used to complete the ‘PNLP1’, which is the monthly malaria surveillance and logistics report. Comprising two essential components, it first documents cases of uncomplicated and severe malaria, diagnosed clinically without testing and with testing (rapid diagnostic test (RDT) or microscopy) according to age groups (0–5 years and over 5 years). In its second component, it provides information on stock management for malaria control materials. The PNLP1 is transmitted to HZs and subsequently incorporated into the District Health Information System, V.2 (DHIS2).

In public health literature, quasi-experimental designs have been extensively applied to routine data to evaluate large-scale health interventions. The ARIMA model, an interrupted time series (ITS) analysis developed by Box *et al*,^29^ has been widely used to evaluate the effectiveness of various public health interventions.^30–32^ Despite its robustness, ITS remains vulnerable to biases, particularly history bias, which threatens validity and limits causal inference.^33^ Alternative methodologies include difference-in-differences with propensity score weighting,^11^ which effectively controls for both observed confounders and time-invariant unobserved heterogeneity but requires the parallel trends assumption and may be sensitive to violations of this key identifying assumption.^34^ Recent studies have also used Bayesian spatio-temporal frameworks to evaluate the impact of SMC using routine data,^35^ providing the benefits of incorporating prior knowledge, quantifying uncertainty through posterior distributions and naturally handling spatial and temporal correlation structures, but these approaches require careful specification of priors, can be computationally demanding and may present challenges in model interpretation and validation for stakeholders unfamiliar with Bayesian inference.^36^ Compared with these methods, the generalised linear mixed model (GLMM) not only accounts for hierarchical data structures and random effects while handling non-normal outcome distributions^37^ but is also relatively straightforward to fit and interpret. However, GLMMs can become computationally intensive and may struggle with convergence issues when dealing with multiple levels of hierarchy, complex random effects structures or sparse data.

This study aims to evaluate the effect of SMC on uncomplicated and severe malaria among children under 5 years of age when delivered through routine programmes by community health workers in northern Benin HZs using a generalised linear mixed-effects model.

## Methods

### Country profile

Benin’s health system has a three-tiered pyramid structure: central (strategic), intermediate (12 departmental directorates) and peripheral (34 operational HZs). The country features distinct climate zones, with northern Benin having a dry tropical climate. This region experiences a unimodal rainy season (June to September), which delivers about 75% of its annual rainfall^38^ and coincides with a peak in malaria transmission. Epidemiological data show that this 4-month period accounts for most malaria cases in children under 5. Consequently, northern Benin was designated a priority for SMC deployment. The implementation of SMC began in 2019 in two HZs and was systematically expanded to six HZs by 2021, following a phased geographical rollout^39–43^ (online supplemental figure 1).

10.1136/bmjph-2025-004335.supp1 Supplementary data

### SMC implementation approach

The SMC intervention delivers monthly SP-AQ treatment (single SP plus 3-day AQ course) to children under 5 during rainy months as recommended by the WHO.^6^ It is delivered through a community-based approach using paired administrators, that is, a technical health worker partnered with an assistant worker such as a village chief or representative. Exclusion criteria include severely ill children, those with presumptive malaria, known allergies to SMC drugs, or recent amodiaquine use. All six SMC-treated HZs eventually received SMC, but uptake was phased: Malanville-Karimama (MK) and Tanguiéta-Matéri-Cobly (TMC) from 2019; Kandi-Gogounou-Ségbana (KGS) and Banikoara (BNK) added in 2020; Natitingou-Toucoutouna-Boukoumbé (NTB) and Kouandé-Kérou-Péhunco (2KP) added in 2021. All HZs received four rounds annually. In 2019–2021, rounds occurred July to October across all active zones. In 2022, all six zones received four rounds, but with staggered timing: KGS, MK and BNK received theirs July to October, while TMC, NTB and 2KP received theirs June to September.

### Selection of intervention and control groups

Benin’s health system comprises 34 HZs. Based on the WHO eligibility criteria, 15 of these zones are eligible for SMC.^44^ Among them, six HZs are currently implementing SMC,^39–43^ and are categorised as the intervention group. The remaining nine eligible zones have not yet received SMC and were thus considered as eligible to be part of the donor pool for the control group. To control for confounding effects, Djougou-Copargo-Ouaké (DCO) HZ, one of the nine remaining HZs, was excluded from the donor pool because it received indoor residual spraying (IRS) in 2019, which typically begins in May and potentially overlaps with SMC implementation (June/July),^45^ making it potentially different from other HZs in terms of transmission patterns. Synthetic control methods^46^ were implemented to construct counterfactual for the SMC-treated HZs. For each treated unit i, donor weights $W^{*}=\left(w_{1}^{*},\;\ldots ,w_{J}^{*}\right)`$ were selected to minimise the pre-intervention (2017–2018) distance between the treated unit’s characteristics (rainfall, temperature, relative humidity) and a weighted average of donor pools, subject to $w_{j}\geq 0$ and $\sum_{j=1}^{J}w_{j}=1$. The pre-intervention fit was assessed using the root mean square prediction error (RMSPE), defined as:

$$RMSPE_{i}=\;\sqrt{\frac{1}{T_{0}}\sum_{t=1}^{T_{0}}{\left(Y_{it}-\;\sum_{j=1}^{J}w_{j}^{*}Y_{jt}\right)}^{2}}$$

where $T_{0}$ is the number of pre-intervention months, $Y_{it}$ is the observed incidence in treated HZ i at time t and $\sum_{j=1}^{J}w_{j}^{*}Y_{jt}$ is the synthetic control outcome. Lower RMSPE values indicate better alignment between the treated unit and its synthetic counterpart. A map showing the SMC-treated HZs and the selected control HZs is presented in online supplemental figure 2.

### Data sources and covariates

Malaria case data covering January 2017 to December 2022 were obtained from Benin’s DHIS-based national health repository, where health facilities report monthly data aggregated at district, HZ and departmental levels. As routinely collected HMIS data were used, no formal sample size calculation was performed; the sample reflected data availability, yielding six intervention and eight control HZs over 72 monthly observations. The study examined two outcomes: confirmed uncomplicated and severe malaria. Confirmed uncomplicated malaria is defined as symptomatic malarial parasitaemia confirmed by a positive diagnostic test result (either rapid diagnostic test or microscopy) in a patient presenting with fever, headache or other malaria-related symptoms, but without severe signs or vital organ dysfunction.^47 48^ Severe malaria is defined as a confirmed malaria infection with signs of severe illness or vital organ dysfunction requiring hospitalisation.^47 49^ The analysis focused exclusively on children under 5 years, excluding cases in individuals aged 5 years and older and pregnant women. Incidence rates for both uncomplicated and severe malaria were calculated per 1000 inhabitants by dividing confirmed case counts by the total population specific to each group residing in the respective HZs.^50^ To control potential confounding factors, facility reporting rates were incorporated to adjust for surveillance disparities across HZs that could otherwise bias intervention effect estimates. Because of the well-documented effect of climate factors, specifically temperature, rainfall and recently relative humidity^51^ on malaria transmission,^52^ they were also included as covariates. Daily climate data (temperature, relative humidity and precipitation) were obtained from the NASA POWER repository^53^ for all HZ centroids in Benin for 2017–2022. Daily values were aggregated monthly (means for temperature and humidity, sums for precipitation) to align with the malaria case series. Optimal lags (1–5 months) per HZ and climate variable were identified via cross-correlation with under-5 malaria cases. The lag yielding the highest absolute correlation coefficient was selected to construct dynamic climate covariates:

$$X_{it}^{dynamic}=\;X_{i,t-l_{i}^{*}}$$

where $X_{it}$ represents the climate variable (precipitation, temperature or humidity) for HZs i  at month t, and $l_{i}^{*}$ is the HZ-specific optimal lag (1–5 months) maximising $\left|\mathrm{cor}\left(Y_{i,t},X_{i,t-l}\right)\right|$, with $Y_{it}$ denoting under-5 malaria cases.

### Statistical analysis

To evaluate SMC’s impact, separate negative binomial mixed-effects models (log link, accounting for overdispersion) were fitted for each outcome. Each model included temporal period (pre-SMC/post-SMC), intervention status (SMC vs non-SMC), dynamic climatic covariates (precipitation, residualised temperature and humidity), facility reporting rates, Long-lasting insecticidal nets (LLIN) coverage and under-5 population as an offset. Random intercepts for HZ and HZ-month interaction accounted for baseline and seasonal heterogeneity. The model can be represented as follows:

$$\begin{array}{l} \log(Y_{it})=\log(N_{it})+\beta_{0}+\beta_{1}{Time}_{t}+\beta_{2}{SMC}_{t}\\ \;+\beta_{3}({SMC}_{t}\times {Time}_{t})+\beta_{4}{Precip}_{it}^{dynamic}\\ \;+\beta_{5}{RH\_resid}_{it}+\beta_{6}{Temp\_resid}_{it}\\ \;+\beta_{7}{Reporting.Rate}_{it}+\beta_{8}{LLIN.Coverage}_{it}\\ \;+\sum_{k=0}^{K}\beta_{9+k}f({month}_{t},d_{t})+u_{i}+v_{it}+\epsilon_{it} \end{array}$$

where $Y_{it}$ represents under-5 uncomplicated (or severe) malaria cases in HZ i at month t; $N_{it}$ is the population offset (children under 5); ${Time}_{t}$ is a binary indicator distinguishing between the pre-intervention and post-intervention period; and ${SMC}_{i}$ is a binary indicator for SMC-treated HZs. A complete description of the model variables and parameters is presented in table 1.

**Table 1:** Description of variables and parameters in the model

Variable/parameter description	
Dependent variable	
*Y_it_*	Total count of uncomplicated (or severe) malaria cases in children under 5 in HZ *i* during time *t* (measured in months).
Independent variables	
*N_it_*	Total population of children under 5 in health zone i at time t (included as an offset).
*Time_t_*	Categorical variable distinguishing between the pre-intervention period (2017–2018) and the post-intervention period (2019–2022).
*SMC_i_*	Binary variable denoting whether a health zone belongs to the intervention group (SMC health zone) or control group (non-SMC health zone).
*SMC_i_*×*Time_t_*	Interaction term quantifying the additional effect of SMC after its implementation.
$Precip_{it}^{dynamic}$	Monthly precipitation per HZs.
$RH\_resid_{it}$	Relative humidity residuals after removing the effect of precipitation, representing the component of humidity independent of rainfall.
$Temp\_resid_{it}$	Temperature residuals after removing the effect of both precipitation and relative humidity, representing the temperature component independent of rainfall and relative humidity.
*Reporting rate_it_*	Completeness of case reporting within health zone *i* at time *t.*
Random effects	
*u_i_*	Random intercept for health zone i ($u_{i}$∼ N (0, σ 2)) to account for geographical heterogeneity in malaria incidence across HZs.^17 96^
*v_it_*	Random deviation for health zone i in month t, capturing month-specific variability within HZs.
*ε_it_*	Error term
Model parameters	
*β*_0_	Intercept term representing the baseline log incidence rate.
*β*_1_	Time effects capturing temporal trends between periods.
*β*_2_	Effect of SMC intervention (compared with control group).
*β*_3_	Interaction term representing the differential effect of SMC over time.
*β*_4_	Effect of precipitation on malaria incidence.
*β*_5_	Relative humidity residuals.
*β*_6_	Temperature residuals.
*β*_7_	Effect of health facility reporting rates on malaria incidence.
*β*_8_	Effect of LLIN on malaria incidence.
*β*_9_ to *β*_13_	Joint effect of the 5° natural spline for seasonality (monthly trends) on malaria incidence.

HZ, health zone; LLIN, Long-lasting insecticidal nets; SMC, seasonal malaria chemoprevention.

To account for delayed climatic effects on malaria transmission, HZ-specific optimal lags (1–5 months) for precipitation, temperature and relative humidity were identified by selecting time delays showing the strongest absolute correlations with under-5 malaria cases,^54^ capturing biologically plausible delays in mosquito breeding and parasite development. Given natural correlations among climate variables in tropical environments, a sequential residualisation approach was employed to eliminate multicollinearity. Precipitation was retained as the primary driver of mosquito breeding; relative humidity was residualised on precipitation; and temperature was residualised on both precipitation and residualised humidity. Results of this orthogonalisation are presented in online supplemental figure 3. Seasonal malaria patterns were modelled using natural cubic splines for months of the year, selected through Akaike Information Criterion (AIC) optimisation to capture non-linear temporal trends while preventing overfitting.^55 56^ Negative binomial regression was selected over Poisson after AIC comparison and confirmation of overdispersion using Pearson residuals^57^ (online supplemental figure 4). From the mixed-effects negative binomial regression model, incidence rate ratios (IRRs) with 95% CIs and p values were estimated. The control group IRR was calculated as the SMC group IRR, and their ratio quantified the differential SMC effect, a ratio below 1 indicating a protective effect. CIs for the ratio were obtained by exponentiating the 95% CI for, assuming approximate normality of the log-ratio. Statistical significance was set at p<0.05. All analyses were conducted in R,^58^ using the glmmTMB^59^ and splines^60^ packages.

### Patient and public involvement

None.

## Results

### Intervention and control group

SMC impact was evaluated by comparing intervention HZs against control HZs. Both groups were eligible for SMC and shared similar climatic characteristics, that is, precipitation, temperature and relative humidity, along with comparable health facility reporting rates (table 2). Intervention HZs were larger geographically, contained more health facilities and had greater total populations than control HZs. However, the proportion of children under 5 remained approximately equivalent between groups (table 2), ensuring comparable baseline malaria risk profiles. Synthetic control fits for the six SMC-treated HZs are summarised in table 3, with pre-intervention RMSPE ranging from 5.2 to 31.9 cases per 1000 population. Five of the six HZs demonstrated good fit (RMSPE <10), confirming adequate resemblance to their synthetic counterparts during the pre-intervention period. 2KP achieved the best fit (RMSPE=5.20; 14.2% of mean baseline incidence, indicating minimal prediction error relative to observed baseline levels), while Banikoara showed the poorest (RMSPE=8.84; 50.9%). TMC’s highest absolute RMSPE (31.9) reflected its substantially higher baseline incidence rather than poor model fit. Overall, the shared climatic conditions, equivalent under-5 proportions and strong synthetic control fits support a methodologically sound comparison of SMC impact between groups.

**Table 2:** Summary of the study: health districts’ characteristics in the pre-intervention period

Background characteristics	Intervention group	Control group
Total surface area (km^2^)	48 127	33 926
Average monthly rainfall (mm) (95% CI)	203.71 (173.07 to 234.34)	212.38 (184.86 to 239.90)
Average monthly temperature (°C) (95% CI)	25.85 (25.65 to 26.06)	25.10 (24.99 to 25.22)
Average monthly relative humidity (%) (95% CI)	81.68 (80.12 to 83.25)	85.69 (84.83 to 86.56)
Total population	3 733 558	3 664 173
Population of children under 5		
Total	664 102	616 214
Proportion (%)	17.8	16.8
HFs		
Total	174	135
Proportion of reporting HFs	77.5	95.6

HFs, health facilities.

**Table 3:** Pre-intervention fit quality of synthetic control models for the six SMC-treated HZs (2017–2018)

Health zones	RMSPE (per 1000)	Mean baseline incidence (per 1000)	RMSPE (% of baseline)
Kouandé-Péhunco-Kérou	5.2	36.6	14.2
Kandi-Gogounou-Ségbana	5.6	18.3	30.9
Malanville-Karimama	6.6	15.7	42.4
Natitingou-Boukoumbé-Toucountouna	7.1	35.9	19.9
Banikoara (ZS)	8.8	17.4	50.9
Tanguiéta-Cobly-Matéri	31.9	71.9	44.3

HZs, health zones; RMPSE, root mean square prediction error; SMC, seasonal malaria chemoprevention; ZS, Zone Sanitaire.

### Trends of uncomplicated and severe malaria-confirmed cases by group and year

Routine surveillance data from 2017 to 2022 were analysed to compare malaria trends in intervention and control HZs before and after the 2019 introduction of SMC in Benin (online supplemental table 1). For uncomplicated malaria, pre-intervention incidence was similar between the control (mean 43.8 cases/1000 and 95% CI 31.8 to 56) and intervention (46.5 cases/1000, and 95% CI 30.0 to 62.9) groups. After SMC implementation (2019–2023), trends diverged. In control HZs, incidences fluctuated, peaking at 61.2 cases/1000 in 2022, with a post-intervention mean of 50.2 cases/1000 (95% CI 36.4 to 64.0). In contrast, intervention HZs experienced an initial drop following SMC rollout in 2019 (from 46.5 to 41.4 cases/1000), and the mean post-intervention incidence was 48 cases/1000, representing a 7.0% reduction compared with the 54.28 cases/1000 observed in control zones over the same period, in absolute terms, this corresponds to 6.3 fewer uncomplicated malaria cases per 1000 children under 5. A significant baseline difference existed for severe malaria, with intervention HZs having a much higher pre-intervention incidence (5.78 cases/1000, 95% CI 3.09 to 8.47) compared with controls (1.46 cases/1000, 95% CI 0.78 to 2.15). Following SMC, severe malaria cases in intervention HZs declined by 45% from their pre-intervention mean to 3.20 cases/1000 (95% CI 2.36 to 4.03), while control HZs remained stable at 1.79 cases/1000 (95% CI 1.53 to 2.05) corresponding to 2.58 fewer severe malaria cases per 1000 children in intervention HZs relative to their pre-intervention baseline. These findings demonstrate a substantial reduction in severe malaria burden associated with SMC implementation.

### Effect of SMC on malaria burden

After adjusting for seasonality and other covariates, the incidence of uncomplicated malaria in the intervention group HZs was unchanged when the 6 months post-SMC was compared with the 6 months pre-SMC (IRR: 1.00, 95% CI 0.87 to 1.14). Over the same interval, incidence in the control group rose by 26% (IRR: 1.26, 95% CI 1.12 to 1.42). The resulting adjusted IRR between the two groups was 0.79 (95% CI 0.65 to 0.98; p=0.008), indicating a statistically significant 21% lower rate of uncomplicated malaria in the intervention group (table 4). Severe malaria showed an even stronger divergence: incidence fell by 39% in the intervention group (IRR: 0.61, 95% CI 0.43 to 0.77) but increased by 30% in the control group (IRR: 1.30, 95% CI 1.01 to 1.66). The ratio of these IRRs was 0.47 (95% CI 0.30 to 0.74, p<0.001), demonstrating a substantial and statistically significant differential trend in severe malaria between the groups (table 4).

**Table 4:** Effect of SMC on incidence of uncomplicated and severe malaria cases: Results of the negative binomial mixed-effect models

Period	Mean incidence rate (1000 persons-months)		Control IRR (95% CI)	Intervention IRR (95% CI)	IRR ratios (%)(95% CI)	P value
	Control HZs (n=6)	Intervention HZs (n=6)				
Uncomplicated malaria						
Before	43.80	46.46	1	1	1	0.004
After	50.84	47.62	1.25 (1.11 to 1.41)	0.97 (0.85 to 1.11)	78 (63 to 96)	
Severe malaria						
Before	1.46	5.77	1	1	1	1.03e−06
After	1.78	3.45	1.26 (0.97 to 1.63)	0.59 (0.44 to 0.79)	47 (30 to 74)	

*Before=pre SMC period (2017–2018); after=post SMC period (2019–2022). IRRs are adjusted for precipitation, relative humidity, temperature, number of reporting health facilities, LLIN coverage and random effects for HZs and month-within-HZ. IRR ratio=intervention IRR/control IRR, representing the differential effect of SMC.

HZs, health zones; IRR, incidence rate ratio; LLIN, Long-lasting insecticidal nets; SMC, seasonal malaria chemoprevention.

## Discussion

This study evaluated SMC effectiveness in Benin using 5 years of routine HMIS data, demonstrating significant reductions in malaria burden compared with control areas, with differential impacts on uncomplicated and severe disease.

### Routine data use in malaria intervention impact evaluation

Routine surveillance data have emerged as a compelling alternative to randomised controlled trials for evaluating public health interventions,^61^ offering health facility-level malaria cases across multiple consecutive years, a temporal dimension valuable for impact assessment.^27^ Historically underused due to data quality concerns,^62–64^ their use has been facilitated by evaluations of National Health Information Management System (NHMIS)^65^ and assessments of factors impairing data quality.^66 67^ The adoption of electronic reporting systems such as DHIS2 in several countries, including Benin, has enhanced comprehensiveness and timeliness.^27^ Initiatives such as monthly synthetic reports in Tanzania^68^ and monthly data validation sessions in Benin^69 70^ have further improved data completeness and accuracy, supporting their increased use in malaria risk studies^68 71^ and evaluations of malaria control interventions.^49 54 72 73^ Nevertheless, data processing and cleaning remain necessary, and internal validity concerns persist despite these improvements. Incorporating climatic variables, reporting rates and appropriate analytical methods helps achieve more realistic impact estimates,^27^ but several HMIS-specific limitations merit explicit acknowledgement. Passive case detection captures only children seeking care at health facilities, meaning differential care-seeking behaviour between groups could bias effect estimates. Additionally, residual under-reporting or differential reporting quality between groups cannot be fully ruled out despite adjustments. Finally, unmeasured confounding may persist. Therefore, while our approach adjusts for confounders, not all possible sources of bias could be eliminated. Results should be interpreted as programmatic effectiveness estimates under real-world conditions rather than as causal effects from a controlled trial.

### Effectiveness of SMC in reducing malaria burden

Substantial evidence supports SMC effectiveness at regional^12 74 75^ and national levels.^14 76–80^ Using Benin’s routine malaria data after 5 years of implementation, this study documented 21% and 53% lower incidence rates for uncomplicated and severe malaria, respectively, in intervention versus control groups. These findings align with previous studies. In Burkina Faso, mixed-effects negative binomial regression reported 32% and 27% reductions in uncomplicated and severe malaria incidence.^49^ A quasi-experimental study in Mali showed SMC reduced confirmed and severe malaria cases by 61% and 72%, respectively.^78^ Elsewhere, uncomplicated malaria incidence growth was 9.7 percentage points lower in SMC districts, while severe malaria declined 17.2 percentage points faster.^81^ In South Sudan, 82% lower odds of RDT-confirmed malaria episodes were found in SMC versus control areas,^77^ and in Chad, SMC was associated with a 20% reduction in both suspected and confirmed malaria incidence.^72^ Despite methodological differences precluding direct comparison of effect magnitudes, all findings consistently demonstrate a positive impact of SMC on malaria burden when deployed through national malaria control programmes.

### Differential impact on uncomplicated versus severe malaria

The observed difference between moderate protection against uncomplicated malaria (21% reduction) and stronger protection against severe malaria (53% reduction) warrants further examination. This pattern differs from some previous studies, such as Kirakoya-Samadoulougou *et al*’s^49^ work in Burkina Faso, which found similar magnitudes of effect across both disease manifestations. Several factors may explain this differential impact in the Benin context. Health system reported biases likely play a significant role. Severe malaria cases typically result in hospitalisation and are more comprehensively documented within the health information system, while uncomplicated cases may be inconsistently reported across facilities. Recent Demographic and Health Survey (DHS) data from Benin^82^ showed low care-seeking behaviour and diagnostic testing rates for children under 5 with fever in SMC implementation areas, which could result in under-reporting of uncomplicated cases. In addition, the RDTs for malaria that health workers routinely use^83^ may have lacked sufficient sensitivity to detect low psarasitaemia and asymptomatic infections,^84^ potentially contributing to the underreporting of uncomplicated malaria cases.

### Disparity in severe malaria incidence between study groups

**Figure 1:**
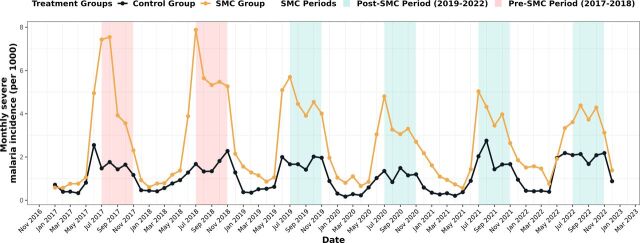
Monthly trends of uncomplicated malaria incidence in health districts before and after SMC implementation. SMC, seasonal malaria chemoprevention.

**Figure 2:**
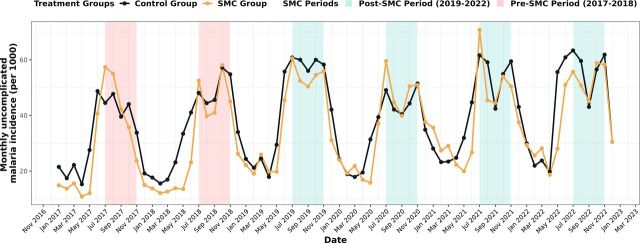
Monthly trends of severe malaria incidence in health districts before and after SMC implementation. SMC, seasonal malaria chemoprevention.

Unlike the incidence of uncomplicated malaria, which remained similar between the intervention and control groups (figure 1), severe malaria incidence was notably higher in the intervention group, particularly during the pre-intervention period (figure 2). This discrepancy poses a substantial challenge to the comparability of the study cohorts. Further analysis of severe malaria incidence trends by HZ (online supplemental figure 5) revealed that the 2KP HZ consistently exhibited higher incidence rates reaching four to five cases per 1000 compared with other HZs, which generally showed rates at or below 2 per 1000. The elevated incidence observed in figure 2 appears largely driven by the inclusion of 2KP in the intervention group. Several factors may explain the higher burden of severe malaria in 2KP. Historically, this HZ has experienced greater malaria transmission intensity than others. Entomological studies conducted in Benin have classified Kerou and Pehunco (two out of the three districts comprising 2KP) as areas of high and medium malaria transmission, respectively. Environmental conditions such as soil type and vegetation index may have also contributed to higher transmission in the region.^85^ Moreover, a high prevalence of malnutrition among children under 5 has been documented in the 2KP HZ.^86^ Malnutrition is known to compromise immune function, increasing both susceptibility to malaria and the likelihood of severe disease outcomes.^87 88^ It may also impair drug metabolism, potentially reduce the efficacy of antimalarial treatment and heighten the risk of reinfection.^89 90^ Given these factors, the elevated incidence in 2KP likely had a substantial influence on the overall results. However, re-running the analysis excluding the 2KP HZ showed very similar results (online supplemental table 2), suggesting that the conclusions remain robust.

### Increased uncomplicated malaria incidence

Uncomplicated malaria cases increased over time in both groups. Beyond waning SMC efficacy, several alternative explanations warrant consideration. First, improved care-seeking behaviour may have contributed to higher reported case counts. Benin’s most recent DHS 2022 data show that the proportion of febrile children under 5 for whom care was sought rose from 42.9% (2011–2012) to 53.1% (2017–2018), suggesting this increasing trend, which predated SMC implementation, could have contributed to higher reported uncomplicated malaria cases. Additionally, COVID-19 communication efforts during the study period may have further promoted care-seeking to some extent, although a study conducted in rural southern Benin^91^ found minimal pandemic-related impacts on health-seeking behaviour. Third, changes in diagnostic practices or RDT availability may have influenced case identification, with the proportion of febrile children tested fluctuating from 17.3% (2011–2012) to 17.7% (2017–2018). Importantly, these factors would likely affect both groups equally, reflecting broader health system trends rather than SMC-specific effects. The interaction term (ratio of IRRs) accounts for shared temporal trends, isolating the differential effect attributable to SMC. Therefore, while uncomplicated malaria incidence increased in both groups, the sustained protective effect of SMC remains evident after accounting for these shared dynamics.

### Implementation considerations and seasonal patterns

Our results indicate that the effect of SMC on uncomplicated malaria is not maintained: incidence drops at first but rises again in later years, whereas the reduction in severe malaria persists despite some year-to-year variation. This rebound could reflect implementation gaps, waning drug efficacy, emerging resistance or shifts in transmission dynamics, all of which merit further investigation. Recent measurements of SMC coverage reported decreasing adherence with each subsequent dose despite reported good overall acceptability of SMC.^92^ This might suggest underlying issues with field implementation. Adverse events, including vomiting, fever and diarrhoea, have been documented^93^ and contribute to either non-acceptance or SMC discontinuation when not adequately managed.^92^ The analysis also revealed pronounced seasonal periodicity in both uncomplicated and severe malaria, with incidence consistently peaking between June and October. This makes it less likely that the observed lack of sustained effectiveness is due to suboptimal timing of implementation. Furthermore, the persistence of seasonal patterns despite SMC suggests that while the intervention reduces the overall burden, it does not fully disrupt the underlying seasonal transmission dynamics.

### Implications for National Malaria Control Programmes

Our findings support the success of past SMC implementation and therefore advocate for the continued implementation of SMC in Benin. The substantial reduction in severe malaria underscores the public health value of this intervention, even if its impact on uncomplicated malaria appears more modest. In the current context of critical malaria funding cuts, these evaluations are essential for informing resource allocation decisions. By quantifying the differential impact of SMC on severe versus uncomplicated malaria, our results provide evidence to prioritise funding toward interventions that avert the most severe outcomes. When funding decisions must be made under constraint, demonstrating that SMC reduces severe malaria cases, the most clinically and economically burdensome form of the disease, offers a clear rationale for prioritising this intervention over others with unproven or smaller effects on severe outcomes. Furthermore, such evaluations help advocate for maintaining funds to continue implementing SMC where it is currently deployed or provide evidence to seek alternative funding sources to sustain malaria control gains in those settings. The demonstrated value of these analytical approaches underscores the imperative for NMCPs to integrate data analytics specialists within their Monitoring & Evaluation (M&E) frameworks, representing a paradigm shift toward evidence-based decision-making that is particularly critical during Mid-Term Reviews.^94^ Embedding modelling capacity enables programmes to harness routine surveillance data, an advantage that becomes even more critical as traditional surveys (eg, Malaria Indicators Survey (MIS), DHS) face increasing discontinuation due to funding constraints.^95^ By doing so, NMCPs can maintain robust evidence generation, optimise intervention targeting and ensure programmatic adaptability and sustained effectiveness even under resource-constrained conditions. Despite these positive results, several considerations warrant attention by the NMCP. First, the observed differences in impact between severe and uncomplicated malaria cases may shadow implementation gaps and deficits in community surveillance that require further investigation. Second, while these results demonstrate the overall effectiveness of SMC, potential disparities in impact across HZs warrant further studies and understanding the drivers of these variations are crucial for programme optimisation. Third, addressing potential implementation gaps, including declining adherence to subsequent SMC doses and management of adverse events is essential to ensure that the gains achieved are sustained and further improved.

### Strengths and limitations

To our knowledge, this is one of the first studies to evaluate SMC impact using routine malaria case data among children under 5 since its implementation in Benin. The study adopted a robust mixed-effects negative binomial regression approach, accounting for overdispersion and the hierarchical structure of the dataset. Random effects at the HZ level controlled unobserved heterogeneity across HZs and months. Internal validity was strengthened by adjusting for time-varying environmental confounders (precipitation, relative humidity and temperature) including their zone-specific lag effects on malaria dynamics. Comparability between intervention and control HZs further reinforced internal validity. Model performance evaluation confirmed good predictive accuracy and successful capture of seasonal malaria patterns across most HZs (online supplemental figures 6 and 7). The use of routine data for impact evaluations presents inherent challenges regarding internal validity, completeness and potential bias in effect estimates, necessitating caution when interpreting analytical findings. To address these concerns, thorough data quality checks for completeness and timeliness were performed following established routine data processing and cleaning methods described by.^68^ Importantly, all health facilities with incomplete reporting, defined as having at least three consecutive missing months per year, were excluded. There are many assumptions that are typically made to conduct any analysis. Our analysis required several important assumptions. Zone-specific SMC administrative coverage frequently exceeded 100% and lacked precision, while effective coverage estimates were only available from a single post-campaign study conducted in 2023.^20^ Consequently, actual SMC coverage could not be adjusted for, and coverage was assumed relatively homogeneous across intervention zones, with SMC administration as the only systematic difference between groups. Other potentially important indicators, such as access to health services and treatment-seeking behaviour, would have also enhanced the internal validity of our routine data. Any time-varying unmeasured confounders that differentially affected intervention and control zones after 2019 could further bias our estimates. Although we excluded the DCO HZ due to IRS exposure, other unmeasured concurrent interventions may remain. Consequently, our findings should be interpreted as strong evidence of association rather than definitive proof of causation. Statistical methods for evaluating public health interventions have inherent limitations: they cannot capture indirect effects such as herd immunity, struggle to model long-term dynamics like waning immunity or resistance development and cannot simulate counterfactual scenarios or fully account for population heterogeneity. Dynamical models address these gaps by explicitly modelling transmission mechanisms, stratifying populations and projecting long-term impacts, enabling assessment of trade-offs, strategy optimisation and prediction of unintended consequences such as drug resistance. Statistical and dynamical models are therefore complementary, with the former providing empirical inputs and the latter integrating them into a mechanistic framework for broader policy insights.

## Conclusion

This study provides robust evidence that SMC effectively reduces malaria burden in Benin, with particularly strong protection against severe disease. The differential impact on uncomplicated versus severe malaria underscores the importance of evaluating multiple outcomes and conducting such studies at national scale to optimise implementation and resource allocation. In the current context of declining malaria funding, these findings justify past investments and advocate for sustained funding commitments to inform evidence-based policy decisions in malaria control.

## Data Availability

Data may be obtained from a third party and are not publicly available. Data from routine HMIS/DHIS2 are not publicly available and were obtained with a request from the National Malaria Control Programme of Benin. Restrictions apply to the availability of these data, and permission can be obtained with a reasonable request from the Ministry of Health.
