# Supplementary material for: Assessing the effectiveness of seasonal malaria chemoprevention on malaria burden among children under 5 in northern Benin: a statistical modelling approach
Source: BMJ Public Health. 2026 Jul 27;4(3):e004335. doi: 10.1136/bmjph-2025-004335 (PMC13410709; doi:10.1136/bmjph-2025-004335)
Supplement: Supplementary data [file bmjph-4-3-s001.pdf]

SUPPLEMENTARY MATERIAL

Supplementary Figure 1. Geographical and Temporal evolution of the implementation of SMC in Benin.

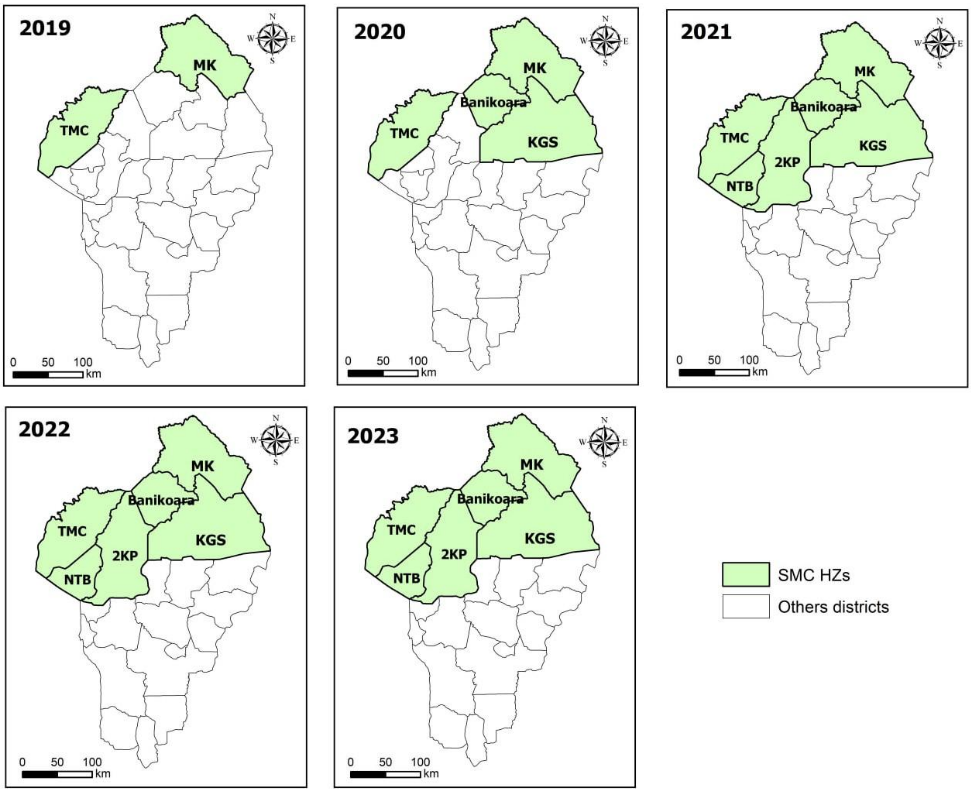

Supplementary Figure 2. Map of the selected HZs: intervention group in green and control group in pink.

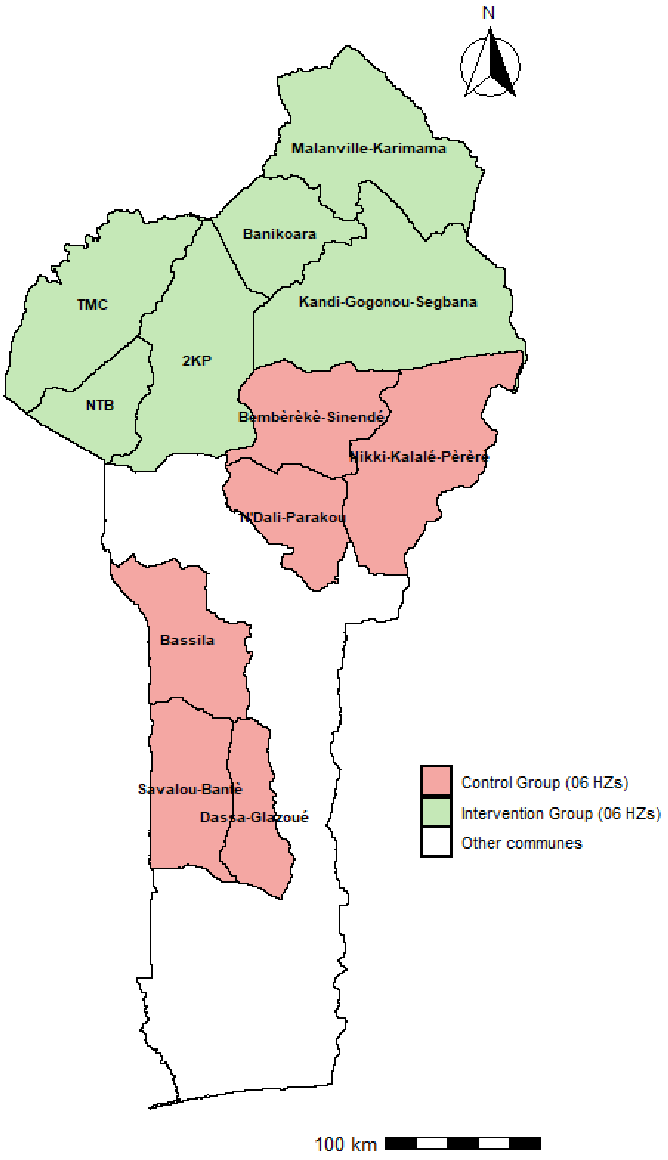

Supplementary Figure 3. Climate Variable Orthogonalization.

To eliminate multicollinearity among the dynamic climate covariates (precipitation, relative humidity, and temperature), a sequential residualisation approach was employed. Precipitation was retained with its full interpretation. Relative humidity was residualised on precipitation, and temperature was residualised on both precipitation and humidity.

The resulting correlation matrix confirmed successful orthogonalisation:

Variable	precip_dynamic	rh_resid	temp_resid
precip_dynamic	1.00	7.645947e-18	-1.286790e-17
rh_resid		1.00	~0.00
temp_resid			1.00

All off-diagonal correlations were effectively zero (on the order of 10^{-17}), indicating that the three climate covariates are mutually uncorrelated. This confirms that multicollinearity has been successfully removed, allowing each variable's independent contribution to malaria transmission to be estimated without bias.

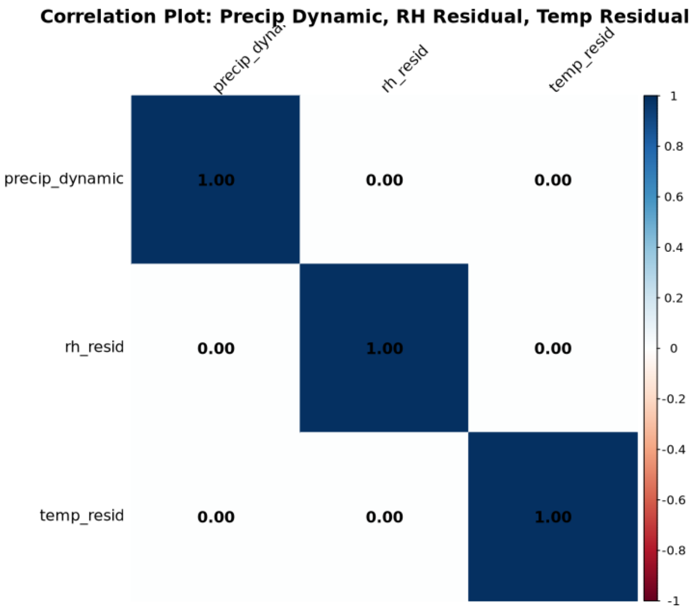

Sonounameto RC, *et al. BMJPH* 2026; 4:e004335. doi: 10.1136/bmjph-2025-004335

BMJ Publishing Group Limited (BMJ) disclaims all liability and responsibility arising from any reliance placed on this supplemental material which has been supplied by the author(s)

ssSupplementary Figure 4. Pearson residuals plotted against fitted values for both negative binomial mixed-effects models: Uncomplicated Malaria Model (A) and Severe Malaria Model (B)

The residuals versus fitted values plot (Supplementary Figure 5) showed random scatter around zero with no systematic pattern, further supporting model adequacy. The lowess smooth (green line) remained close to the reference line (red) across fitted values, and most residuals fell within the ±2 bounds (blue dashed lines), indicating no substantial outliers or misspecification.

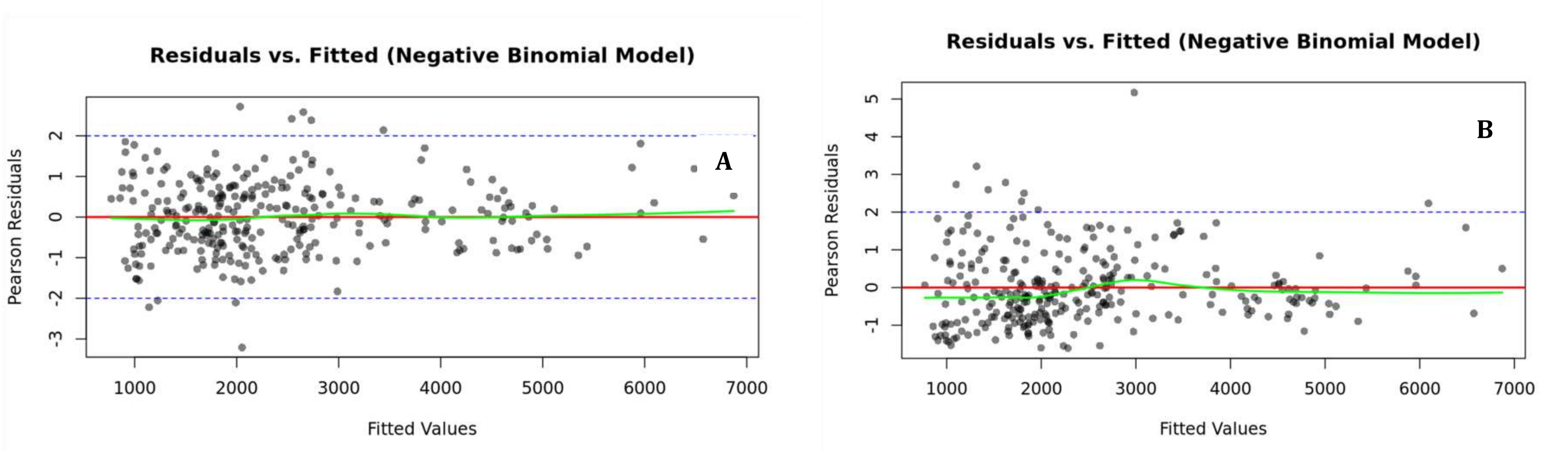

Supplementary Figure 5. Monthly trends of severe malaria incidence per HZs from 2017 to 2022.

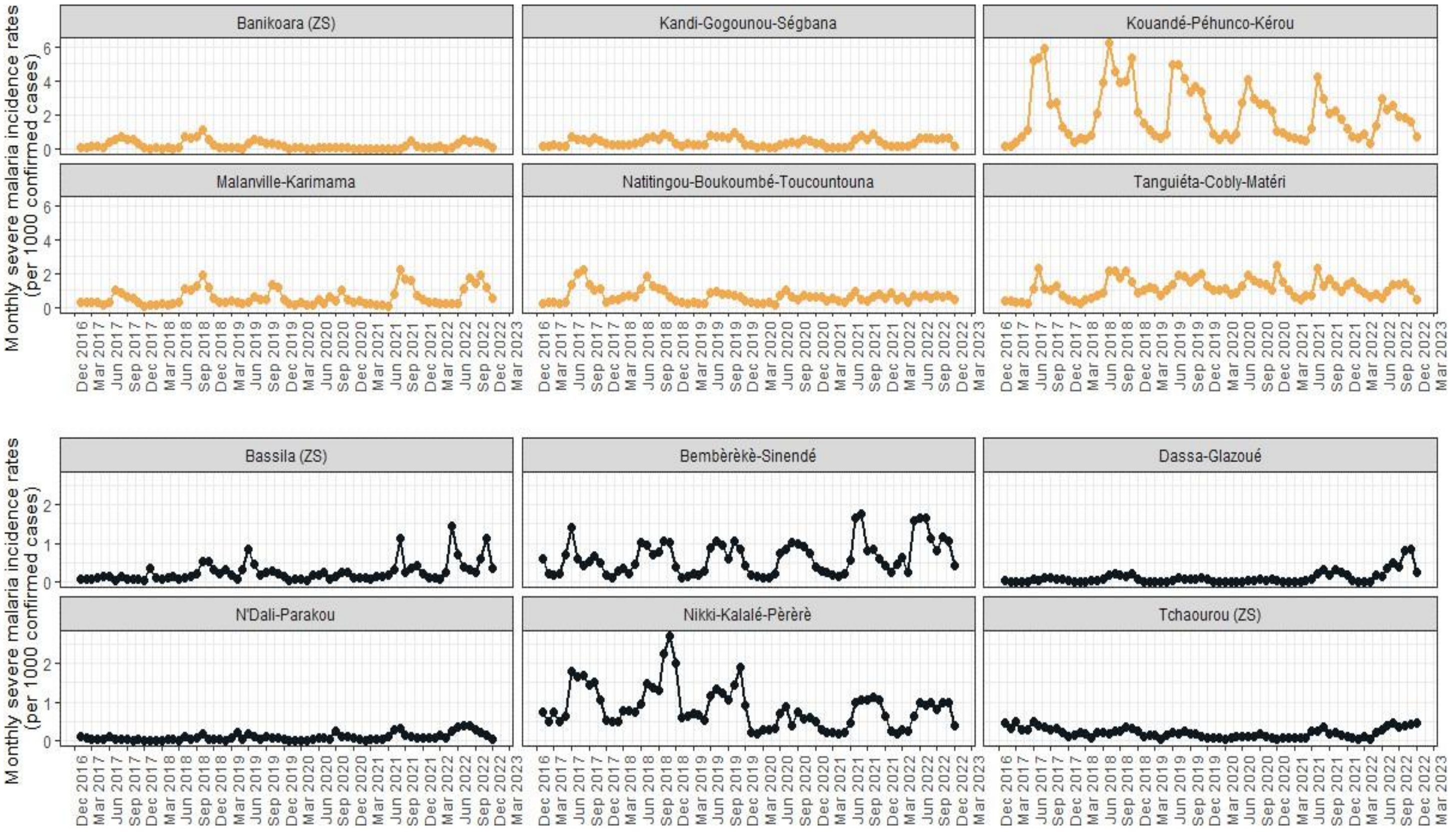

Supplementary Figure 6. Model Performance Assessment: Predicted vs Observed Uncomplicated Malaria Cases by Health Zone, 2017-2022.

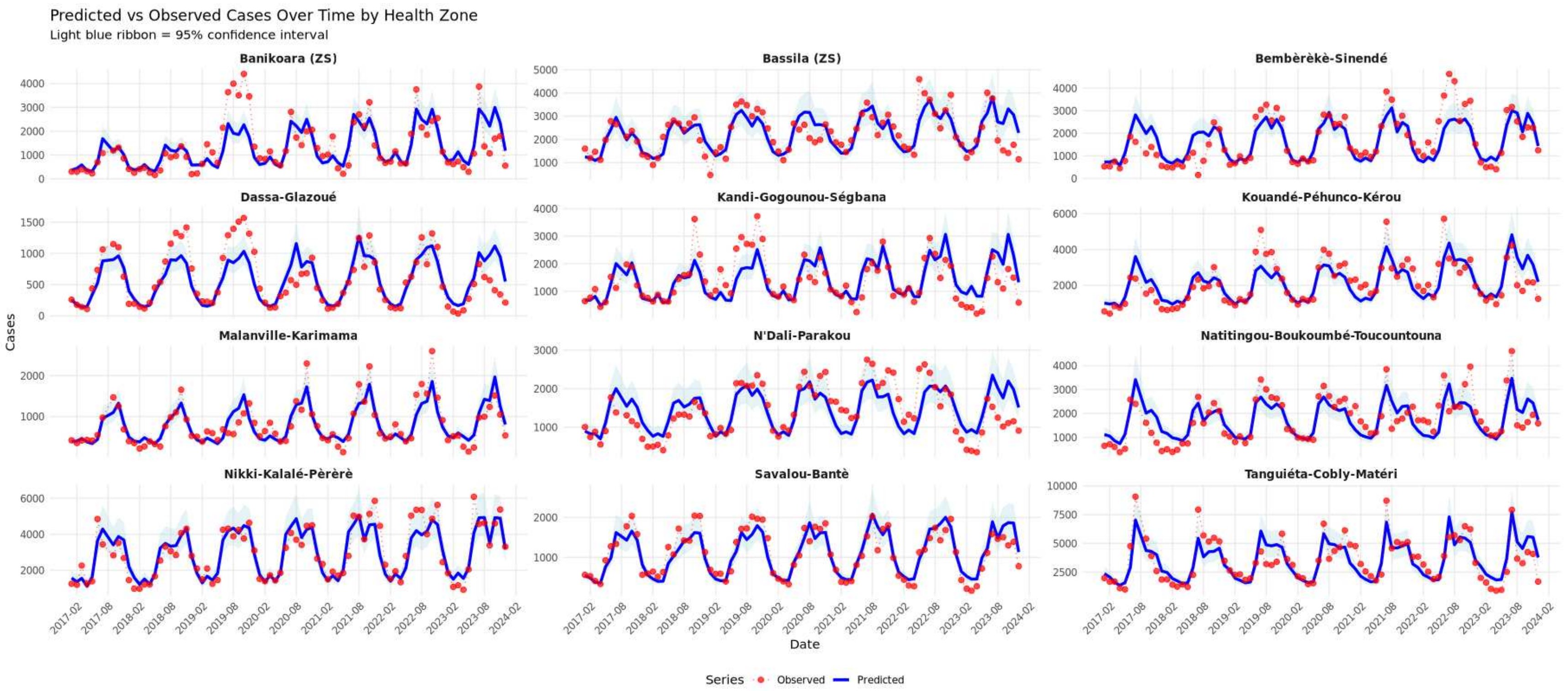

Based on your DHARMa diagnostic results, the model demonstrates strong statistical properties: the non-significant KS test (p = 0.172) confirms the residuals follow a uniform distribution, indicating no systematic bias in the model structure. The dispersion test (p = 0.872) shows the negative binomial family appropriately handles overdispersion without under- or over-dispersion issues, while the non-significant outlier test (p = 0.72) verifies no extreme observations are unduly influencing the results. Together, these diagnostics confirm that the model's fixed effect estimates are reliable, the standard errors are trustworthy, and the inference drawn from coefficients such as the significant type×time interaction and environmental predictors is statistically valid.

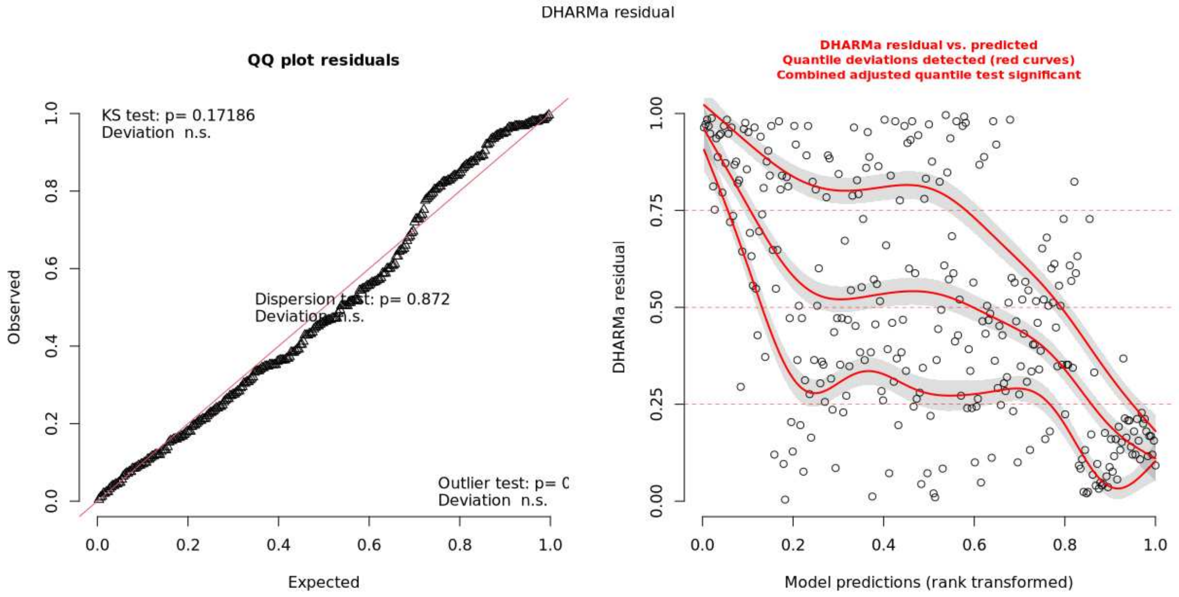
DHARMa residual
QQ plot residuals
KS test: p= 0.17186
Deviation n.s.
Dispersion test: p= 0.872
Deviation n.s.
Outlier test: p= 0
Deviation n.s.
Observed
Expected
DHARMa residual vs. predicted
Quantile deviations detected (red curves)
Combined adjusted quantile test significant
DHARMa residual
Model predictions (rank transformed)

Supplementary Figure 7. Model Performance Assessment: Predicted vs Observed Severe Malaria Cases by Health Zone, 2017-2022.

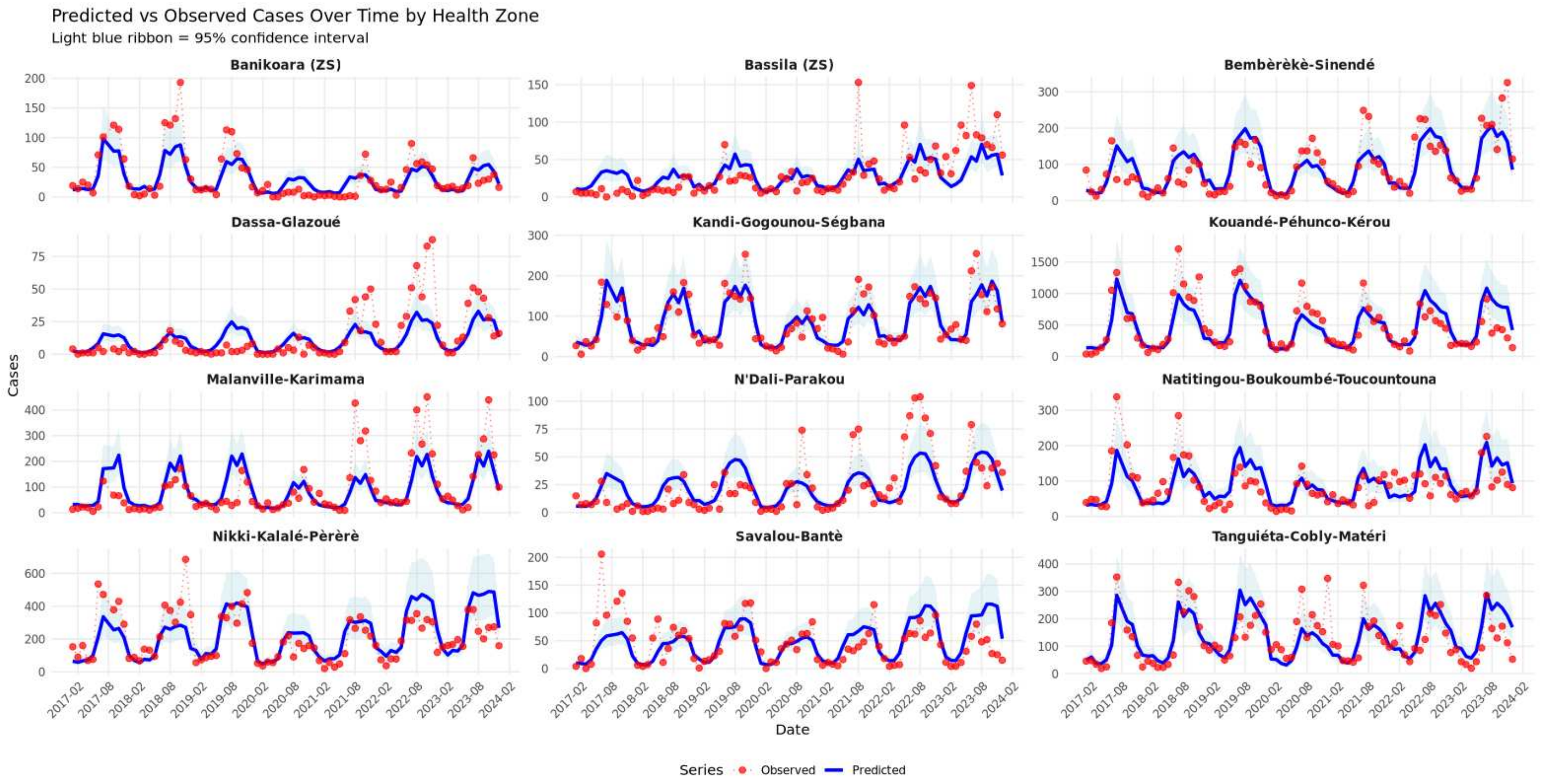

The model performs well in two key areas: the non-significant KS test (p = 0.22) confirms the residuals follow a uniform distribution without systematic bias, and the non-significant dispersion test (p = 0.86) indicates the negative binomial family appropriately handles overdispersion. However, the significant outlier test (p = 0.002) reveals the presence of extreme observations that warrant attention, and the residual-vs-predicted plot shows quantile deviations suggesting room for improved model specification. While the coefficient estimates remain reliable, these diagnostic flags indicate that investigating potential outliers and nonlinear relationships could further enhance model fit.

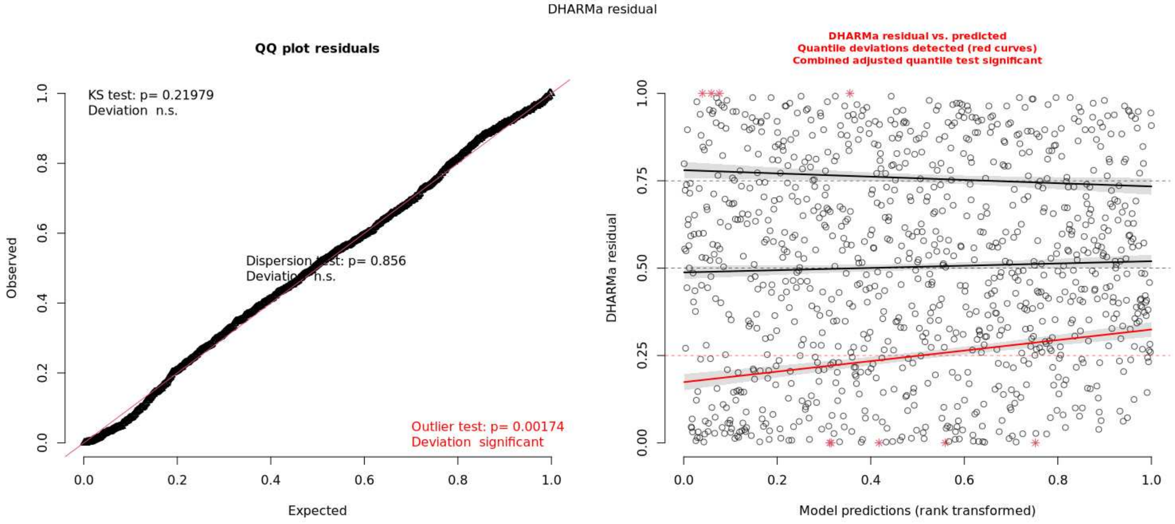

Sonounameto RC, *et al*. *BMJPH* 2026; 4:e004335. doi: 10.1136/bmjph-2025-004335

Supplementary Table 1. Number of confirmed total, uncomplicated and severe malaria cases by group and year among children under 5 years, 2017–2022.

	Control Group		SMC Group	
Year	Uncomplicated malaria	Severe malaria	Uncomplicated malaria	Severe malaria
2017	43.88 [31.73–56.02]	1.57 [0.79–2.35]	46.54 [29.10–63.98]	5.53 [2.87–8.19]
2018	43.71 [29.83–57.60]	1.35 [0.77–1.94]	46.38 [30.86–61.90]	6.02 [3.30–8.74]
2019	58.93 [43.12–74.73]	1.69 [1.07–2.31]	41.40 [17.51–65.28]	2.44 [1.20–3.67]
2020	43.95 [34.14–53.77]	1.21 [0.78–1.64]	45.95 [27.35–64.55]	1.62 [0.74–2.50]
2021	53.08 [39.61–66.54]	1.93 [1.27–2.59]	52.54 [38.62–66.46]	4.19 [2.39–5.99]
2022	61.15 [43.99–78.30]	2.10 [1.62–2.59]	52.27 [41.67–62.86]	4.10 [2.63–5.56]
2023	38.81 [26.98–50.63]	2.03 [1.43–2.63]	41.23 [28.17–54.29]	3.63 [2.39–4.87]

Supplementary Table 2. Effect of SMC on incidence of uncomplicated and severe malaria cases: Results of the negative binomial mixed-effect models without the 2KP HZ in the intervention group.

Period	Mean Incidence Rate (1000 persons-months)		Comparison IRR (95% CI)	Intervention IRR (95% CI)	IRR ratios (%) [95% CI]	p-value
	**Control HZs (n = 6)**	**Intervention HZs (n = 6)**				
Uncomplicated malaria						
Before	44.18	48.94	1	1	1	0.002
After	50.84	47.45	1.26 [1.12–1.43]	0.95 [0.84–1.07]	75 [60–94]	
Severe malaria						
Before	1.34	3.35	1	1	1	<0.001
After	1.78	2.52	1.40 [1.10–1.80]	0.60 [0.46–0.78]	43 [27–68]	

Sonounameto RC, *et al. BMJPH* 2026; 4:e004335. doi: 10.1136/bmjph-2025-004335
